# Sport resumption and quality of life after surgical correction of anomalous origin of a coronary artery from the opposite sinus

**DOI:** 10.3389/fcvm.2023.1099544

**Published:** 2023-04-04

**Authors:** Camille-Océane André, Amir Hodzic, Charles Dolladille, Pascale Maragnes, Cynthia Cousergue, Pierre Ollitrault, Jimmy Sayegh, Emré Belli, Fabien Labombarda

**Affiliations:** ^1^ Department of Pediatrics, CHU de Caen-Normandie, Caen, France; ^2^ Department of Cardiology, Normandie University, UNICAEN, CHU Caen-Normandie, Inserm Comete, GIP Cyceron, Caen, France; ^3^ Pharmacoepidemiology Unit, Department of Pharmacology, Normandie University, UNICAEN, CHU Caen-Normandie, Caen, France; ^4^ Department of Cardiology, CHU de Caen-Normandie, Caen, France; ^5^ Department of Pediatric and Adult Congenital Heart Diseases, Marie Lannelongue Hospital, Groupe Hospitalier Saint Joseph Reference Center of Complex Congenital Heart Diseases M3C, Le Plessis Robinson, France; ^6^ Department of Cardiology, Normandie University, UNICAEN, CHU Caen-Normandie, UR 4650 PSIR, Caen, France

**Keywords:** sport, quality of life, coronary artery, congenital disease, cardiac surgery

## Abstract

**Objectives:**

We sought to assess the resumption of sport, exercise performances, and quality of life (QoL) in adults and children after surgical repair of anomalous coronary arteries originating from the opposite sinus (ACAOS).

**Materials and methods:**

Patients who underwent surgical repair for ACAOS between 2002 and 2022 were retrospectively identified. Information about sports activity and exercise performance based on metabolic equivalents of task (METs) calculated at the last exercise stress test, were collected. QoL was assessed using age-appropriate questionnaires (Paediatric QoL Inventory, cardiac module version 3.0 for patients <18 years; SF-36 QoL Inventory for adults). Patients' METS and patients’ QoL-scores were compared to reference population using the Wilcoxon test.

**Results:**

45 patients were enrolled (males 71%, adults 49%, anomalous right coronary 84%). Median age at surgery was 15 years; median follow-up after surgery was 2.3 years [4 months–12 years]. All post-operative exercise stress tests were normal, METs and VO2 max patients' values did not differ from healthy children or adults (Exercise intensity: 12.5 ± 4.7 vs. 13.4 ± 2 METS, *p* = 0.3; VO2 max: 43.6 ± 16.6 vs. 46.9 ± 7 ml/kg/min, *p* = 0.37). For adults, QoL—scores were similar between ACAOS patients and controls. For children, there was no significant difference between the study patients' scores and those of the reference population, except for physical appearance proxy-report (*p* = 0.02).

**Conclusion:**

In our study, the practice of sports, exercise stress testing and QoL were not adversely affected after ACAOS repair.

## Introduction

Among the group of congenital coronary artery anomalies, anomalous origin of coronary artery from the opposite sinus (ACAOS) with an interarterial course of the ectopic proximal vessel represents a subgroup associated with the greatest potential for clinical consequences, specifically sudden death, in teenagers and young adults during strenuous physical activity (1–5). The estimated prevalence of these high-risk ACAOS in the general population ranges from 0.1% to 0.6% (6). According to international guidelines, surgical correction is strongly recommended for ACAOS presenting with symptoms or diagnostic evidence consistent with coronary ischaemia attributable to the anomalous coronary artery (7). The treatment for asymptomatic patients, especially those with an abnormal right coronary artery (R-ACAOS) from the opposite sinus, remains controversial (8), and is the most common case observed in the clinical setting (9). ACAOS is the second leading cause of sudden death in young athletes (10) and, because of the undeniable desire to continue intense competitive physical activity, is the most influential factor motivating asymptomatic athletes to undergo surgical correction of the abnormal congenital coronary anomaly. Data on the resumption of sports and exercise in adult and paediatric patients after surgical correction of ACAOS are limited. Additional objective data on the resumption of physical activities after correction for congenital coronary artery anomaly are needed. Therefore, we conducted a retrospective study to assess the resumption of sports and exercise and quality of life (QoL) in adults, adolescents, and children who underwent surgical repair of ACAOS.

## Materials and methods

This study was conducted in accordance with all institutional guidelines related to patient confidentiality and research ethics, including institutional review board approval. Due to the retrospective study design, informed consent requirements were waived. The study protocol was approved by the local ethics and health research committee (CLERS N°2925).

In a first step, we retrospectively identified consecutive adults, adolescents, and children with diagnosed ACAOS who underwent surgical repair from the congenital heart clinic databases of both tertiary Caen University Hospital Center and tertiary Marie Lannelongue Hospital Center. Eligible patients were identified by reviewing patients' medical records in the institution's informatics systems at both centres. Patients with other types of congenital coronary anomalies and patients with associated structural heart diseases were excluded from the study. Data, including patient characteristics, information related to ACAOS, and postoperative exercise performance, were collected. Second, selected patients were contacted to complete QoL questionnaires. All data were entered into a reliable database.

### Postoperative exercise performance

**Figure 1:**
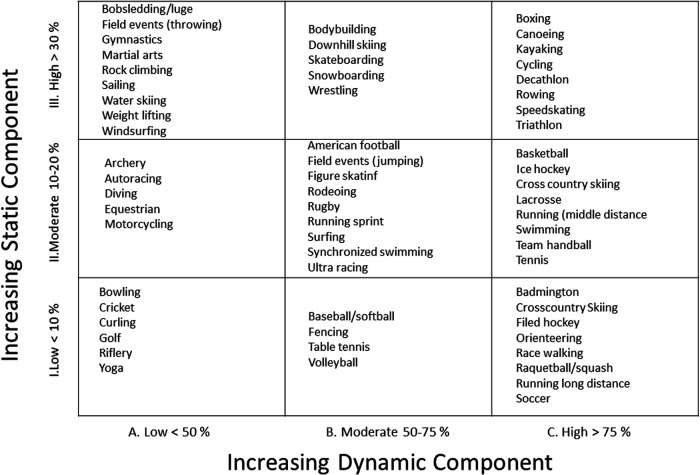
Classification sport based on peak static and dynamic component (12).

Postoperative exercise performance was evaluated based on the exercise stress test (EST) results, time to sport resumption, and level of sport intensity after cardiac surgery. For each patient, the last EST performed during routine cardiac follow-up was recorded in the medical record. Patients exercised to their maximum ability using a cycloergometer or a treadmill. The following criteria were collected: comprehensive EST results (normal/abnormal), arrhythmia (yes/no), blood pressure response (normal/abnormal), metabolic equivalent (expressed in METs) and VO2 max calculated (formula: VO2max = 3.5 × MET; expressed in ml/kg/min). EST results were compared to reference values observed in healthy volunteers (11). Level sport intensity was defined according to the classification of sports based on static and dynamic components (Figure 1) (12).

### Quality of life questionnaires

Patients or parents were called before QoL questionnaires were sent. In case of no response, a second reminder was sent. In the absence of a response after the second reminder, patients were excluded from the QoL analysis. All questionnaires were completed between February 2022 and July 2022.

For patients aged ≤18 years, the PedsQL Paediatric Quality of Life Inventory, cardiac module version 3.0 was used (13). There were separate questionnaires for patients (self-report) and parents (parent-proxy report). The PedsQL 3.0 cardiac module has 5 scales: symptoms (7 items), perceived physical appearance (3 items), treatment anxiety (4 items), cognitive problems (5 items), and communication (3 items). An additional treatment barriers scale (5 items) is embedded in the module to measure adherence issues for patients who take medications. The validity and reliability of the PedsQL cardiac module scales for parent proxy reports for children aged 2 to 18 years and for self-reports for children aged 8 to 18 years were previously reported (14, 15). The questionnaires were divided by age into child (8 to 12 years) and teenager (13 to 18 years) groups. A 5-point scale is used for child self-report and parent proxy reports (0, never a problem; (1) almost never a problem; (2) sometimes a problem; (3) often a problem; and (4) almost always a problem). Items are reverse-scored and linearly transformed to a scale with scores ranging from 0 to 100 points such that higher scores indicate better QoL. PedsQL scores were compared to health-related QoL scores from a reference paediatric population with a mild cardiovascular disease requiring no therapy or effectively treated without surgery (14).

For adults (18 years and past), we utilized the SF-36 QoL Inventory. These consisted of 11 questions regarding physical and psychological dimensions. A total score for each section was generated. The score ranged from 0 (worst functioning) to 100 (best functioning). SF-36 QoL scores were compared to the reference SF-36 QoL scores observed in a general population of young adults aged 25–34 years (15).

### Statistical analysis

The study population was described using means and medians for quantitative variables and with frequencies for qualitative variables. The patients were divided into two groups for statistical analysis: children and adults. Patients' metabolic equivalents of task (METs) were reported and compared to normal values for children and age-matched healthy subjects for adults (11) using the Wilcoxon test.

The PedsQL 3.0 cardiac module scale scores for children were compared with those with a mild cardiovascular disease requiring no therapy or effectively treated without surgery (16) using the Wilcoxon test. Individual item analysis with ranking was performed. No general scale score is available for the cardiac module. SF-36QoL inventory scale scores for adults were compared with those of a healthy adult population (17) using the Wilcoxon test. The statistical analyses were carried out with R, 4.2.1 Windows version software. For all analyses, a *p* value <0.05 was considered statistically significant.

## Results

### Population study

**Figure 2:**
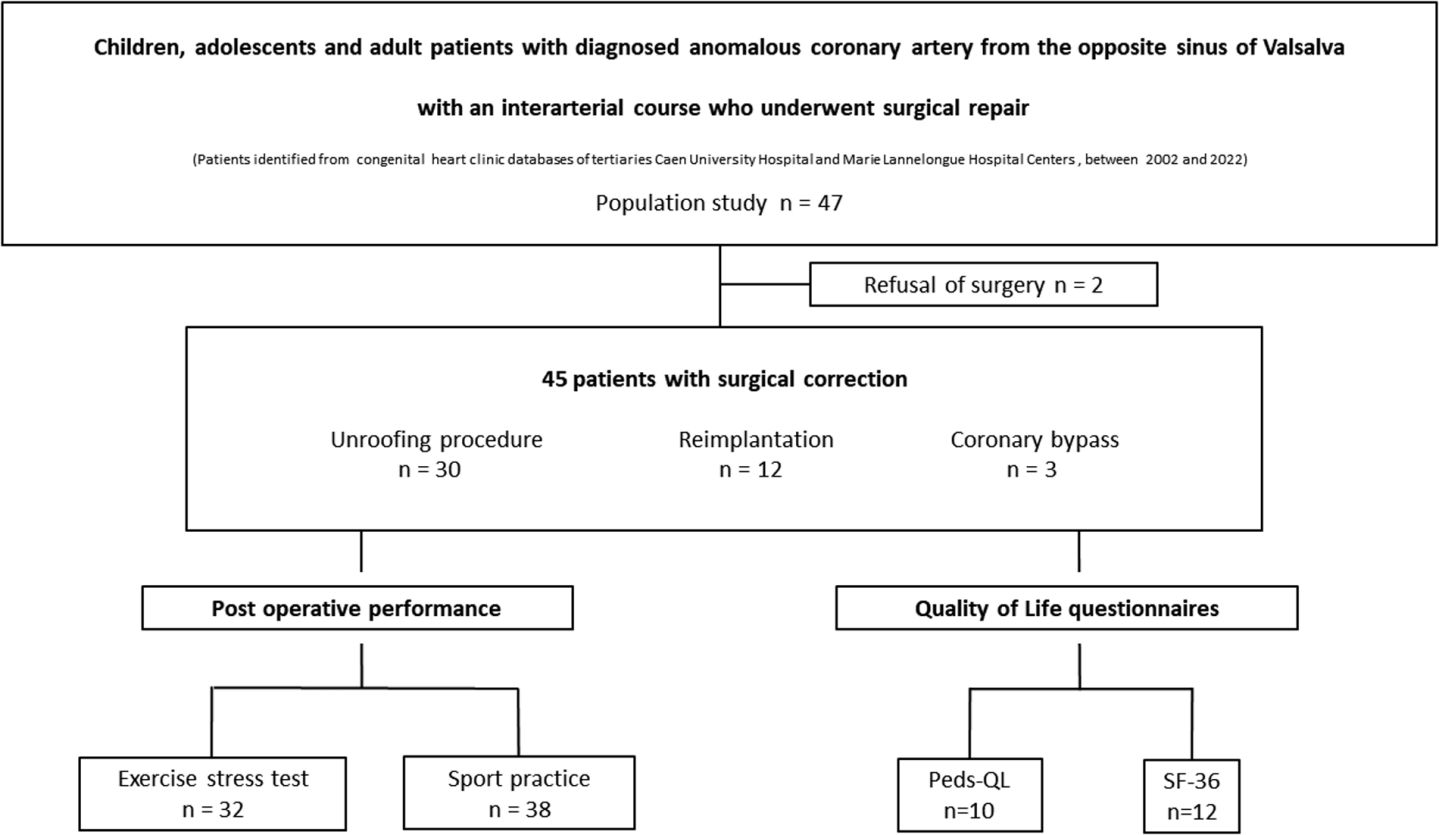
Study flow chart.

Overall, 45 consecutive patients who underwent surgical repair of ACAOS between 2002 and 2022 were enrolled. Among them, 32 (71%) were males, 22 (49%) were adults and 38 (84%) had R-ACAOS. Twenty-nine (64%) had cardiac symptoms at diagnosis, and 3 (7%) had aborted sudden cardiac death. 16 patients were diagnosed fortuitously after a cardiac examination motivated by heart murmur, screening for familial bicuspid aortic valve and screening for athletes with competitive sport activity. Among these asymptomatic patients, 8 patients practised competitive sports. Twenty-six (58%) underwent stress tests preoperatively, two of them presented with inducible myocardial ischaemia detected by nuclear myocardial perfusion imaging. The median age at the time of surgery was 15 years; the median duration of follow-up after surgery was 2.3 years [4 months–12 years]. Intra mural course was confirmed intra operatively in 28 patients (62%). The unroofing procedure was performed in 30 (67%) patients, reimplantation of the ectopic coronary artery was performed in 12 (27%) patients, and 3 (6%) had a bypass. There was no perioperative death; 8 patients (18%) had acute complications, including pericardial effusion (*n* = 7) and haemothorax (*n* = 1). Finally, 1 (2%) patient required reintervention for late complications of ostial patch aneurysm after coronary reimplantation. The characteristics of this group are summarized in Table 1. Complications according to surgical techniques are depicted in Table 2. Among the 45 patients enrolled, 38 patients returned to sports and competitive sports postoperatively. EST results was collected for 32 patients, and 22 patients completed the QoL questionnaires (Figure 2).

**Table 1:** Characteristics of the study population.

	All patients (*n* = 45)	R-ACAOS (*n* = 38)	L-ACAOS (*n* = 7)
**Demographics**			
Age (years)	19 [12–32]	18 [12–31]	17 [11–37]
Male gender	32 (71%)	26 (68%)	6 (86%)
**Presenting symptoms**			
Exertional chest pain	9 (20%)	8 (21%)	1 (14%)
Palpitations	3 (7%)	3 (8%)	0
Syncope	2 (4%)	2 (5%)	0
Acute coronary syndrom	1 (2%)	0	1 (14%)
Sudden cardiac death	3 (7%)	2 (5%)	1 (14%)
No symptoms	16 (36%)	12 (32%)	4 (57%)
Non exertional chest pain	11 (24%)	11 (29%)	0
**Pre-operative myocardial ischemia testing**			
Exercise stress test	12 (27%)	9 (24%)	3 (43%)
Nuclear myocardial perfusion imaging	7 (16%)	6 (16%)	1 (14%)
Stress cardiac MRI	1 (2%)	1 (3%)	0
Stress echocardiogram	6 (13%)	5 (13%)	1 (14%)
None	19 (42%)	17 (45%)	2 (29%)
**Pre-operative inducible ischemia**	0	2*	0
**Type of surgical repair**			
Unroofing procedure	30 (67%)	25 (66%)	5 (71%)
Coronary reimplantation	12 (27%)	12 (32%)	0
Coronary bypass	3 (6%)	1 (3%)	2 (29%)
**Early post operative complications**			
Pericardial effusion	7 (16%)	7 (18%)	0
Hemothorax	1 (2%)	1 (3%)	0
Reintervention**	3 (7%)	3 (8%)	0
**Late post operative complications**			
Pericardial effusion	1 (2%)	1 (2.5%)	0
Coronary patch aneurysm	2 (4%)	1 (2.5%)	1 (1.5%)
Reintervention***	1 (2%)	1 (2.5%)	0

R-ACAOS: anomalous right coronary artery from the opposite sinus of Valsalva with an interarterial course.

L-ACAOS: anomalous left coronary artery from the opposite sinus of Valsalva with an interarterial course.

Data are expressed as number (*n*) and pourcentage (%), except for age expressed as median [25th, 75th percentile].

*2 positive nuclear myocardial perfusion.

**Drainage of pericardial effusion *n* = 2; drainage of hemothorax *n* = 1.

***Coronary patch aneurysm repair *n* = 1.

**Table 2:** Early and late postoperative complications according to surgical technique.

	Unroofing (*n* = 30)	Reimplantation (*n* = 12)	Coronary bypass (*n* = 3)
R-ACAOS	24 (80%)	12 (100%)	1 (33%)
**Early post operative complications**			
Pericardial effusion	5 (17%)	2 (17%)	0
Hemothorax	0	1 (8%)	0
Reintervention*	1 (3%)	2 (17%)	0
**Late post operative complications**			
Pericardial effusion	0	1 (8%)	0
Coronary patch aneurysm	1 (3%)	1 (8%)	0
Reintervention**	1 (3%)	0	0

R-ACAOS: anomalous right coronary artery from the opposite sinus of Valsalva with an interarterial course.

Data are shown as *n* (%).

*Drainage of pericardial effusion *n* = 2; drainage of hemothorax *n* = 1.

**Coronary patch aneurysm repair *n* = 1.

## Postoperative exercise performance

### Exercise stress test

All the EST results were concluded to be normal. No patients had clinical signs of ischaemia, arrhythmia, or abnormal blood pressure adaptation during the EST. Mean delay between ACAOS surgery and EST was 2 years. There was no significant difference in METs and VO2 max between the normal values observed in children or age-matched healthy adults. The results are reported in Table 3.

**Table 3:** Post operative exercise stress test.

	Post operative exercise stress test	Reference value *	*p* value
**Children and adolescent**			
Age (years)	12 ± 3	–	–
Maximal heart rate (% theoretical maximal heart rate)	87 ± 5.6	–	–
Exercise intensity (METs)	12.5 ± 4.7	13.4 ± 2	0.37
VO2 max calculated (ml/kg/min)	43.6 ± 16.6	46.9 ± 7	0.37
**Adults**			
Age (years)	28 ± 13	–	–
Maximal heart rate (% theoretical maximal heart rate)	90 ± 8	–	–
Exercise intensity (METs)	12.2 ± 4	12.4 ± 2.14	0.86
VO2 max calculated (ml/kg/min)	42.9 ± 14.3	43.5 ± 7.5	0.86

METs, metabolic equivalent of task; MHR, maximal heart rate.

*Reference value from (11).

### Resumption and intensity of sport after surgery

**Figure 3:**
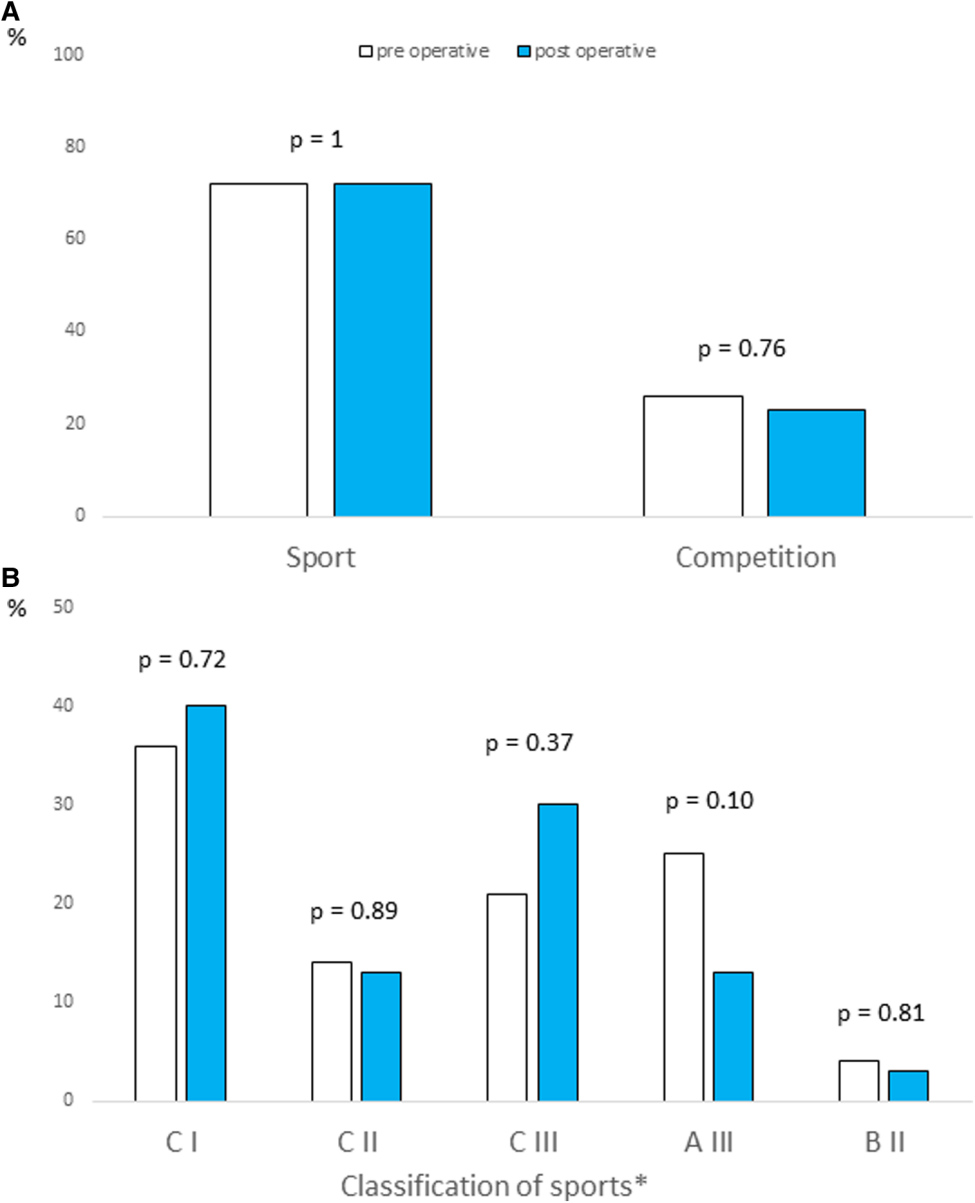
Resumption (panel A) and intensity (panel B) of sport in post operative period. *According to the AHA Guidelines (12).

Information regarding the sport before and after surgery was available for 38 patients. Of those, 10 had competition activities before surgery, 28 had recreational sport; all of 38 patients with sport activities were allowed to return to sports and competition after a normal EST. One patient without symptom decided to stop competition after surgery (Figure 3, panel A). Some patients changed their sports activities, choosing sports with more dynamic and static components (Figure 3, panel B).

## QoL questionnaires

**Figure 4:**
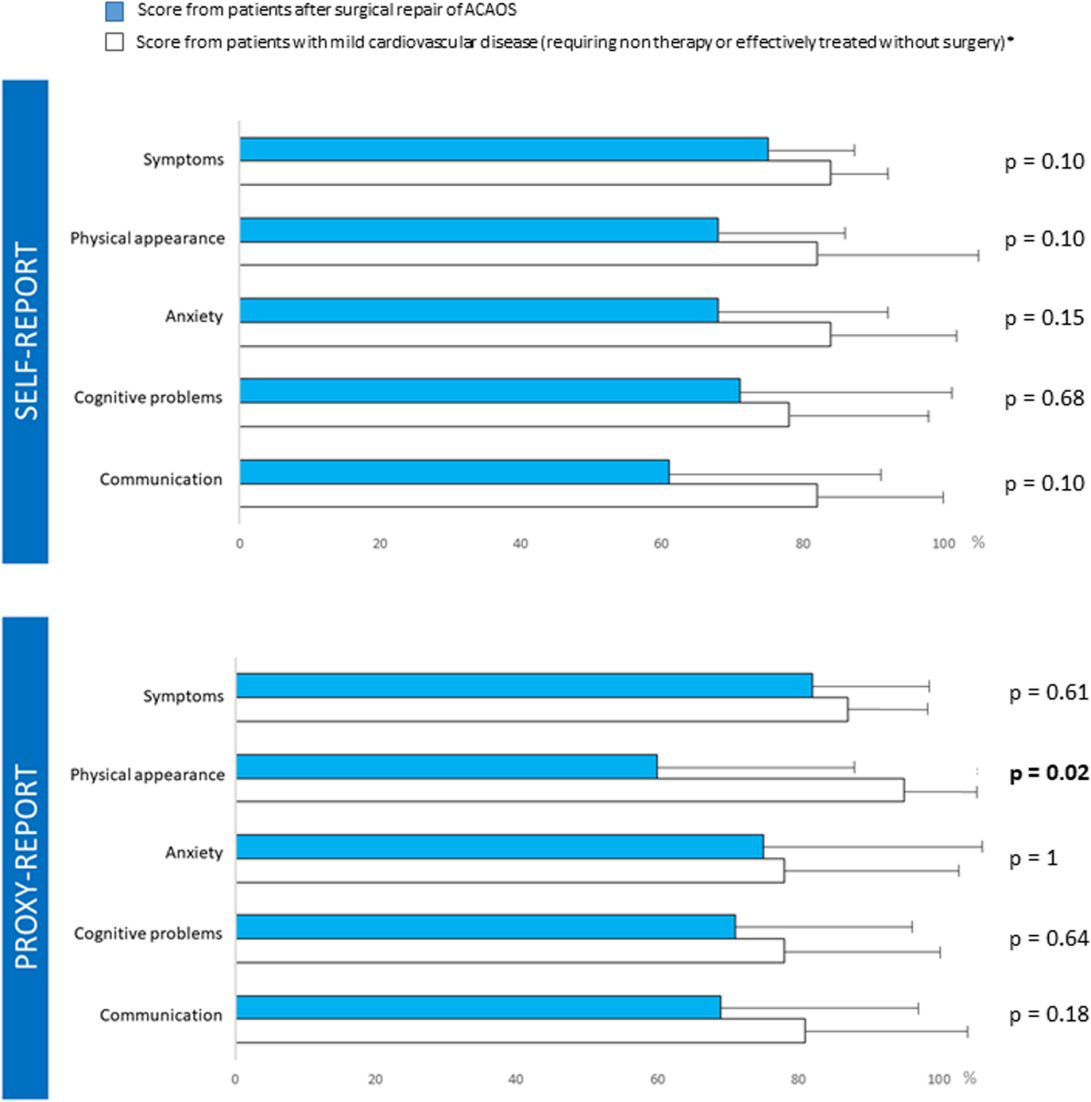
Post operative Ped-Qol questionnaires. ** Score from patients with mild cardiovascular disease (requiring non therapy or effectively treated without surgery) (14).

Ten children (43%) completed an age-appropriate PedsQL 3.0 cardiac model questionnaire after surgical repair. The mean age of patients at the time of PedsQL completion was 12 ± 1.7 years. There was no significant difference between the study patients' scores and those of the reference population with mild heart disease, except for physical appearance proxy-report (*p* = 0.02). The results are depicted in Figure 4.

**Figure 5:**
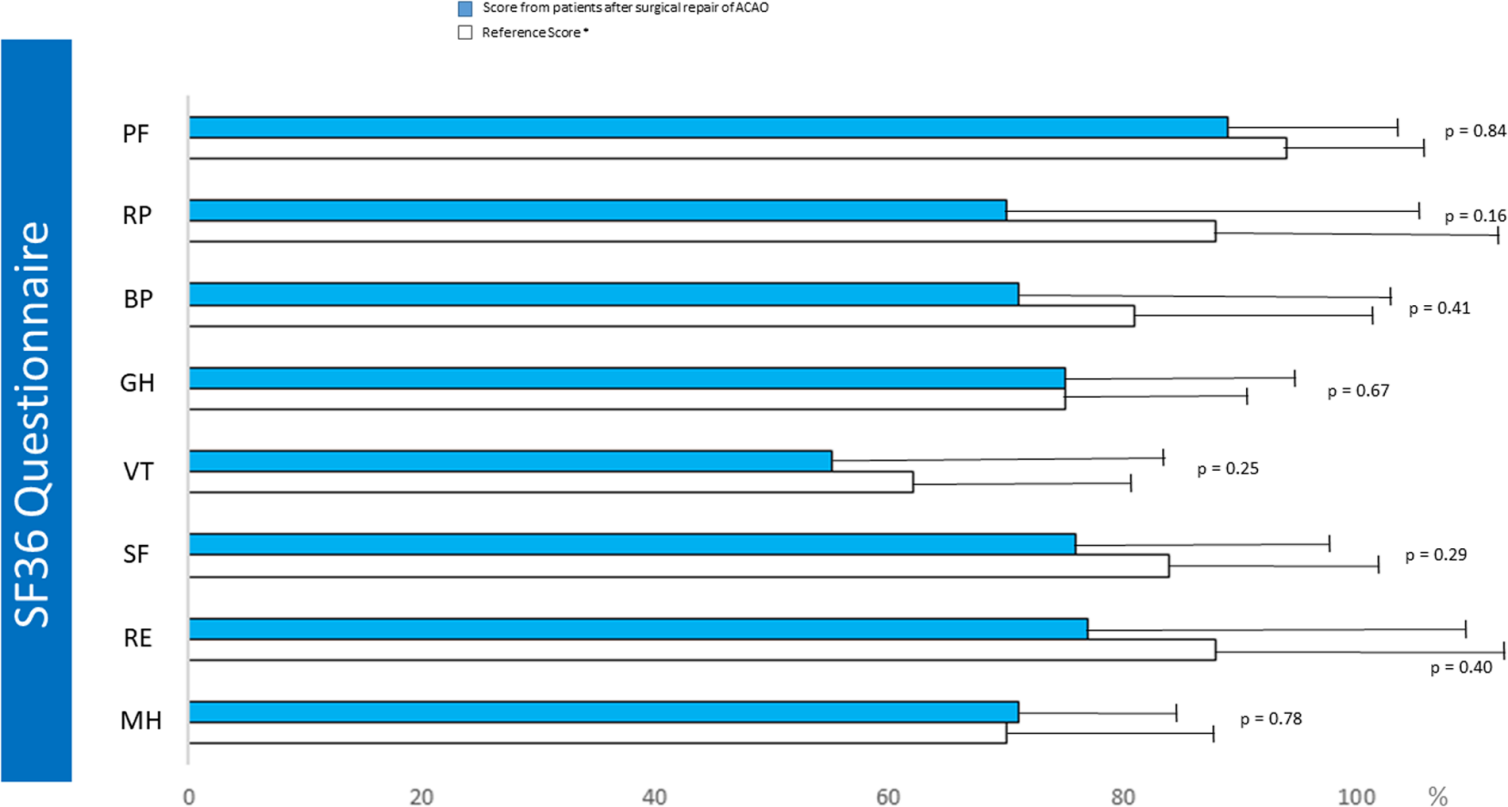
Post operative SF36 QOL questionnaire. PF, physical fonction; RP, restriction due physical status; BP, body pain; GH, global Health; VT, vitality; SF, social fonction; RE, restriction due to emotional status; MH, mental health; HT, health trends; PCS, physical score; MCS, mental score. *Reference score from (15).

Among adult patients, 12 (34%) completed the SF-36 QoL questionnaire (Figure 1). All patients reported a normal QoL after surgical repair for ACAOS, both in physical and psychological dimensions. There was no significant difference in the QoL questionnaire scores between the patients and healthy subjects (Figure 5).

## Discussion

The current study showed that surgery for ACAOS did not impact the resumption of competitive sport activity. The QoL scores of patients who underwent ACAOS surgery were comparable to those of reference populations.

When deciding to recommend surgical correction of ACAOS in athletes, especially in asymptomatic patients, the first object of therapy is to prevent sudden cardiac death during exercise by treating the intramural path of the ectopic coronary artery. Several techniques have been described but, in our experience, the most appropriate surgical technique to address ACAOS is the unroofing procedure of the intramural segment, which is simple, safe and associated with favorable outcomes (18–21). Alternative techniques, including ostioplasty alone, coronary reimplantation with neo-ostial enlargement, or pulmonary translocation, have also shown good results and can be proposed in some cases (22, 23).

The second objective of surgical correction of ACAOS in athletes is to recover a similar level of physical activity with return to competition. Although international guidelines on the management of ACAOS have been recently published, the management of ACAOS remains problematic, mainly in asymptomatic athletes practising a competitive sport given the close link between strenuous exercise and sudden death (24, 25). For athletes, the fear of sport-related sudden death amplified by media coverage, and the strong desire to participate in high-level sports, are important factors motivating the active request for surgical correction of ACAOS, which synonymous with definitive cure. Therefore, the continuation of the same high-level physical activity with the same level of competitiveness is questioned at the diagnosis and as soon as surgical treatment is mentioned. There are almost no data on the return to physical activity or the return to competitive sports after surgical correction of ACAOS in athletes, which motivates us to complete our work. In our study, the practice of competitive sports was not adversely affected by ACAOS repair, as all patients resumed a competitive sport activity after heart surgery. Brothers et al. were the first to demonstrate that patients had normal physical working capacity after ACAOS repair and could perform exercise at the same level before and after surgery (26), which was consistent with our findings. Brothers et al. also reported unexplained mild chronotropic impairment in children and adolescents following ACAOS surgery, which we did not observe in our population. The mean delay between surgery and the EST was longer in our study than in the Brothers' study, which may suggest normalization over the time of postoperative chronotropic changes and may explain our different results. According to the recent AHA/ACC Guidelines, after surgery for ACAOS, patients may return to competitive sports at least 3 months postoperatively if they are asymptomatic and if there are no complex cardiac arrhythmias or evidence of inducible myocardial ischaemia observed during the maximal exercise stress test (27). Therefore, the detection of myocardial ischaemia is important in the preoperative and postoperative periods. Today, the most appropriate stress testing protocol for the detection of myocardial ischaemia in patients with ACAOS remains to be determined (28, 29). In our practice, until now, we supported stress protocols that most closely restore haemodynamic conditions during physical activities and vigorous sports, including EST, myocardial nuclear perfusion imaging with EST, and stress echocardiogram. In another study with longitudinal data, Brothers et al. reported a low incidence of ischaemia detection in a small number of symptomatic patients with ACAOS who underwent a preoperative physical stress test (30), which was consistent with our findings. It is well known that these tests may yield false negative results in patients with ACAOS because of the difficulty in reproducing intermittent ischaemia (31). To attempt to overcome this problem in postoperative evaluation, patients underwent the EST, which included reaching a high physical workload beyond the classical exercise target of 85% of the maximum heartbeat based on the patient's age, as previously suggested (28), although reliable predictive targets have not been validated in this context in any study. Recent data have suggested that cardiopulmonary exercise test has an increased sensitivity for the detection of myocardial ischaemia (31). Finally, stress MRI has become an increasingly interesting and suitable modality for the detection of myocardial ischaemia in both children and adult patients with ACAOS (32, 33). In our practice, we did not use a systemic cardiopulmonary exercise test and stress MRI for ACAOS evaluation, but considering these recent studies, we prefer to use these 2 modalities to detect inducible myocardial ischaemia in our future patients with ACAOS and for cardiac follow-up. Invasive testing were proposed to overcome the limits of non-invasive functional exploration. Invasive testing including intravascular ultrasound and fractional flow reserve with dobutamine stress may be the most adequate tests for the detection of ischemia in ACAOS. Nevertheless, it requires a high level of expertise, it should be performed by ACAOS-experienced interventional cardiologists (34) and it is not feasible in children. Such invasive studies may also play a useful role in patients with abnormal anatomic or functional studies postoperatively (18). However, because of its invasiveness, limited experience to expert centers and absence of guidelines we did not propose for the moment these invasive modalities for the evaluation and the standard follow-up of our patients after ACAOS repair. Considering the lack of long-term data after ACAOS repair, stress tests, including EST or stress MRI, associated with ECG-Holter, to identify potential myocardial ischaemia and arrhythmia, must be repeated yearly in patients who perform competitive sports activities.

A third therapeutic objective of ACAOS repair is to maintain the same QoL considering that most of these children, teenagers and young adults were otherwise in good health and had normal lives before being diagnosed as having ACAOS. The question of QoL in patients after heart surgery has become a key issue. Studies on the QoL of children and adults undergoing surgical treatment for congenital heart disease, including ACAOS, have yielded conflicting results probably due to the different populations assessed and the lack of methodological consistency (35, 36). Although patients' QoL after heart surgery seems to depend on various determinants, 2 factors seem to particularly affect QoL after surgery for congenital heart disease: the severity of the cardiac disorder and the physical performance after correction (37, 38). Amedro et al. reported good QoL in children and teenagers with simple CHD; they also demonstrated a correlation between exercise capacity and QoL (38). It was also reported in a large-scale international study that adults with simple CHD had a good QoL (39). In this study, the impact of functional status on QoL was also emphasized. Our results of good QoL after ACAOS repair are consistent with these previous reports. Contrary to our findings, Agrawal et al. reported a decreased QoL in children and adolescents with ACAOS as perceived by patients and caregivers independently of surgical status (40). In their study most patients were unoperated at the time of the questionnaire which may explain their different results (40). In our study, based on the QoL questionnaire analysis, we did not discern any negative impact of heart surgery experience. As QoL questionnaires were collected after sport recovery, we speculate that recovery of both similar preoperative physical status and competition activity may explain the good QoL scores in patients after ACAOS repair. Proxy experiences may negatively influence the perception of QoL in patients (41). This was not observed in our study, although scores for physical appearance were lower in proxy reports. Finally, in our assessment of QoL in the patient and parents, we failed to detect potential anxiety due to the history of coronary malformation with a risk of sudden death. Our results suggest that either patients or parents no longer perceive the risk of sudden death after the surgical procedure. This feeling of healing, magnified by the return to competition, should not cause patients to stop their long-term cardiac follow-up.

## Limitations

Our study results must be interpreted within the context of its limitations. First, we performed a retrospective study with a small sample size mainly due to the inherent challenges associated with such a rare disease. Second, QoL scores and EST results were not available for all the population due to the retrospective design and typical low response rate for the questionnaire. Third, QoL score and EST were not available for all the population but only for 58% of patients. Fourth, we have no information on the reasons of the patients for changing their sports activity after surgery, in particular we do not know why some patients changed to a sport classification with lower intensity. Fifth, we did not explore the QoL of the adult patients' families. Finally, data on the delay between surgery and return to sport are missing, which prevents us from giving a precise postsurgery recovery time.

## Conclusion

In our study, the practice of competitive sports and QoL were not adversely affected by ACAOS repair.

Our data may help physicians provide information on the ability to return to sport and competition as soon as the diagnosis of ACAOS is confirmed. Further collaborative studies are required to determine the long-term impact of the diagnosis of ACAOS on sport activity and QoL of these young patients.

## Data Availability

The raw data supporting the conclusions of this article will be made available by the authors, without undue reservation.
